# Oral Hygiene and Periodontal Treatment Needs in Adult Patients with Cystic Fibrosis (CF)

**DOI:** 10.3390/healthcare10050766

**Published:** 2022-04-20

**Authors:** Tomasz Hildebrandt, Elżbieta Świętochowska, Agata Trzcionka, Anna Zawilska, Henryk Mazurek, Dagmara Mączkowiak, Mansur Rahnama, Marta Tanasiewicz

**Affiliations:** 1 Department of Conservative Dentistry with Endodontics, Faculty of Medical Sciences in Zabrze, Medical University of Silesia, Plac Akademicki 17, 41-902 Bytom, Poland; thildebrandt@sum.edu.pl (T.H.); azawilska@sum.edu.pl (A.Z.); dagmara.maczkowiak@sum.edu.pl (D.M.); martatanasiewicz@sum.edu.pl (M.T.); 2 Department of Medical and Molecular Biology, Faculty of Medical Sciences in Zabrze, Medical University of Silesia, Ul. Jordana 19, 41-808 Zabrze, Poland; elaswieta@interia.pl; 3 Department of Pneumonology and Cystic Fibrosis, Institute of Tuberculosis and Lung Diseases, Ul. Prof. Jana Rudnika 3B, 34-700 Rabka Zdrój, Poland; hmazurek@igrabka.edu.pl; 4 Department of Dental Surgery, Medical University of Lublin, Ul. Dr W. Chodźki 6, 20-930 Lublin, Poland; mansur.rahnama@umlub.pl

**Keywords:** cystic fibrosis, systemic inflammatory disease, oral hygiene, periodontal treatment need

## Abstract

Cystic Fibrosis (CF) is an autosomal multisystem recessive genetic disease. Patients with Cystic Fibrosis, oral bacteria related to dental and periodontal diseases that can also inhabit the lungs, increases the risk for systemic complications. Our study aimed at assessing oral hygiene status of cystic fibrosis adult patients. The study was conducted on 40 patients diagnosed with CF and 40 healthy participants. The following indices were included: Simplified Oral Hygiene (OHI-S), Approximal Plaque Index (API), Community Periodontal Index of Treatment Needs (CPITN), and a questionnaire. Obtained results proved that the API was 44.63% in the study group, indicating sufficient hygiene, and 37% in the control group, indicating quite good hygiene. Significantly higher OHI-S was found in the study group. It was found based on the analysis of treatment needs that home care and professional instructions on proper oral hygiene were more often needed in the control group compared to CF patients. In conclusion, the obtained API and OHI-S values in adult CF patients were indicative of satisfactory oral hygiene. Periodontal treatment needs assessed based on the CPITN index in patients with CF indicated the need for professional preventive treatments. An interdisciplinary dental care to support oral health could be recommendable in individuals with chronic respiratory diseases such as Cystic Fibrosis.

## 1. Introduction

Dental status is implicated in several systemic inflammatory diseases, such as chronic respiratory diseases, and is a potential focus for systemic infections [1,2]. Cystic Fibrosis (CF) is an autosomal multisystem recessive genetic disease. The disease is caused by mutations of the CF Transmembrane Conductance Regulator (CFTR) gene, which can be found on the long arm of chromosome 7 (7q31.2), and is responsible for coding protein with the same name [2,3]. Completely symptomatic Cystic Fibrosis is characterized by chronic-broncho-pulmonary disease, exocrine pancreatic insufficiency (EPI), and a pathognomonic increase in the concentration of chloride in sweat. Patients affected by the disease show many symptoms in other organs [1].

Treatments for CF include aggressive airway clearance therapy and nutrition support, often including regular high calorie snacks, nutritional supplements, and pancreatic enzyme replacement therapy [4]. In addition to diet and saliva, recurrent courses of antibiotics and multiple surgeries may have adverse effects on tooth development [1]. Especially for severely diseased patients, such as patients with Cystic Fibrosis (PWCF), oral bacteria related to dental and periodontal diseases can also inhabit the lungs, increasing the risk for systemic complications [2].

Same studies showed better oral hygiene, with lower levels of gingivitis and plaque among people with Cystic Fibrosis than controls. Despite this, many studies showed that PWCF had higher levels of dental calculus [5]. Saliva helps protect teeth by buffering against the harmful effects of dietary sugars. However, the CFTR gene also affects the salivary glands of patients with Cystic Fibrosis. Clinical manifestations include salivary gland hypertrophy and structural alterations, especially in the mucus-producing sublingual gland. Enamel defects, predominantly enamel opacities, are more common in CF patients [6,7,8]. Such defects become dentally disabling and would also permit the removal of dental calculus deposits [9]. Molar incisor hypomineralization (MIH) is a special form of the described abnormality in patients with Cystic Fibrosis. It was defined in 2001 by Weerheijm et al. as a ‘hypomineralisation of systemic origin, observed as demarcated, qualitative defects of enamel of one to four first permanent molars (FPMs) frequently associated with affected incisors’ [3,7,10].

The aim of our study was the assessment of oral health in adult patients with CF based on oral hygiene indices—Approximal Plaque Index (API), Simplified Oral Hygiene Index (OHI-S), Community Periodontal Index of Treatment Needs (CPITN)—and a questionnaire.

## 2. Materials and Methods

### 2.1. Materials

The research was performed on 80 patients who were divided into study and control groups [11].

A study group was composed of 40 patients who suffered from disease exacerbation and were treated in Rabka-Zdrój (Institute of Tuberculosis and Lung Diseases, Department of Pneumonology and Cystic Fibrosis). People over 18 years old with a genetically confirmed diagnosis of Cystic Fibrosis (CF) and who agreed to participate in the study were included into the study group. Patients under 18 years of age, who did not agree to take part in the research were excluded from the research.

The control group included 40 patients. Adult patients (over 18 years of age), who were not diagnosed with cystic fibrosis nor with any other general disease, who gave a written consent to participate in the study were included. Younger individuals with diagnosed general diseases and who refused to participate in the study were excluded. Patients from the control group were treated for the first time at the Academic Center of Dentistry and Specialist Medicine in Bytom.

### 2.2. Method

In order to conduct the dental examination, the following dental equipment was used: mouth mirror, dental probe, periodontal probe-calibrated WHO-621, and dental tweezers. Oral hygiene and periodontal therapeutic needs were assessed. The obtained clinical findings were recorded.

#### 2.2.1. An Assessment of Oral Hygiene

Oral hygiene was evaluated using the:

- Approximal Plaque Index (API) according to Lange. It allows for the assessment of oral hygiene in a large population and is useful in the comparative analysis against other clinical parameters. After the inspection of dental plaque using a probe, interdental hygiene was assessed. In quadrants 1 and 3, the presence of bacterial plaque was verified from the aspect of the oral cavity proper, while in quadrants 2 and 4—from the vestibular aspect. The sum of recorded interdental spaces with plaque (“+”) was divided by the sum of all inspected spaces and multiplied by 100 to obtain percentage values.
  API interpretation:
  100–70%: insufficient (poor) oral hygiene;
  70–40%: sufficient oral hygiene; improvement of oral hygiene is needed; 39–25%: quite good hygiene
  <25%: optimal conditions for protection against dental caries and periodontal diseases [12].
- The modified OHI-S according to Green and Vermillion (Simplified Oral Hygiene Index). This index determines both the amount (deposition rate) as well as the type of dental deposits as it consists of a simplified debris index (DI-S) and simplified calculus index (CI-S). Debris and calculus are assessed on 6 dental surfaces (6 teeth):
  - buccal surface of teeth 16 and 26
  - lingual surface of teeth 36 and 46
  - labial surface of tooth 11
  - lingual surface of tooth 31.
  Criteria for classifying debris and calculus are the same:
  0—No debris or calculus present.
  1—Supragingival debris or calculus covering not more than one third of the exposed tooth surface.
  2—Supragingival debris or calculus covering more than one third but not more than two thirds of the exposed tooth surface or the presence of individual flecks of subgingival calculus.
  3—Supragingival calculus covering more than two third of the exposed tooth surface or a continuous heavy band of subgingival calculus.

The obtained values for individual teeth are added and divided by the number of inspected surfaces. The CI-S and DI-S values may range from 0 to 3; and the total OHI-S value ranges from 0 to 6.

The interpretation of OHI-S scores is based on the classification presented by Greene and Vermilion:

0—excellent hygiene
0.1–1.2—good hygiene
1.3–3.0—regular hygiene
3.1–6.0—poor hygiene [12].

#### 2.2.2. Questionnaire

In order to obtain more detailed information regarding oral hygiene habits, participants were asked to fill in a questionnaire. Questions and possible answers were as follows:

1. When have you started to take care of oral cavity hygiene? (as a teenager, in my 20’s, in my 30’s, after 30 years of age)
2. How long does it take you to brush your teeth? (1 min, 2 min, more than 2 min, I don’t know)
3. What kind of toothbrush do you use? (ultra-soft, soft, medium, hard, electronic, manual)
4. What type of the toothpaste do you use? (with fluoride in concentration 1000–1400 ppm, herbal, whitening, for dentinal hypersensitivity)
5. Do you use any other additional products to maintain proper oral hygiene? (dental floss, toothpick, chewing gum, none)
6. Do you use mouthwash? (yes, no)
7. Do you brush your tongue? (yes, no)
8. How often do you visit a dentist? (very rarely, only in case of pain, every 3–6 months)

Periodontal status was assessed using

- The Community Periodontal Index of Treatment Needs (CPITN) according to *Ainamo*. The examination was performed using a calibrated probe (WHO 621) with a blunt tip end allowing for the assessment of the depth of gingival crevices and pockets without traumatizing effects on the periodontium. The examination was performed in sextants, which correspond to the following dental segments: 17–14, 13–23, 24–27, 37–34, 33–43, 44–47. At least 2 teeth must be present in a sextant for it to be scored. If only 1 tooth is present in a sextant, it is included in the adjoining sextant.
  The clinical description of CPI codes is as follows:
  0—Healthy;
  1—Bleeding on probing;
  2—Calculus detected or conditions promoting plaque retention, pockets < 3 mm;
  3—Pockets 3.5–5.5 mm
  4—Pockets > 6 mm.
  Management according to the TN code:
  CPI-0: TN-0 home care;
  CPI-1: TN-1 instructions on proper oral hygiene;
  CPI-2, CPI-3: TN-2 instructions on proper oral hygiene, professional scaling;
  CPI-4: TN-3 complex periodontal treatment [13].

#### 2.2.3. Statistical Analysis

The IBM SPSS Statistics was used for the statistical analyses. The following analyses were performed: basic descriptive statistics, chi-square independence test, Mann–Whitney tests, Spearman correlation coefficient. Data were regarded statistically significant if *p* < 0.05. Results at 0.05 < *p* < 0.1 were considered statistically significant at the level of statistical tendency.

## 3. Results

### 3.1. General Characteristics of Study Participants

A total of 24 women and 16 men were included into the study group. Their mean age was 26 ± 6.46 years. The control group was composed of 25 women and 15 men aged between 18 and 37 (mean age was 24.33 ± 5.11). There was no statistically significant difference between the groups with regard to age (*p* = 0.202) [11].

In the study group, there were four people diagnosed with hypertension, one treated because of heart diseases, eight diagnosed with diabetes. None of these general diseases was observed in the control group.

### 3.2. An Assessment of Oral Hygiene in Adult CF Patients

#### Oral Hygiene Indices

**Figure 1:**
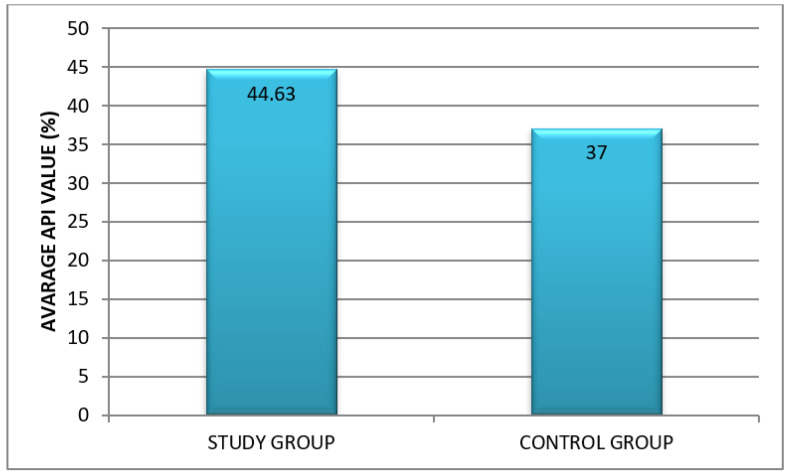
Oral hygiene assessment based on API in the study and control group.

The analysis of Approximal Plaque Index (API) in both groups showed differences at the level of statistical tendency. The API was 44.63% in the study group, indicating sufficient hygiene, and 37% in the control group, indicating quite good hygiene (Figure 1).

**Figure 2:**
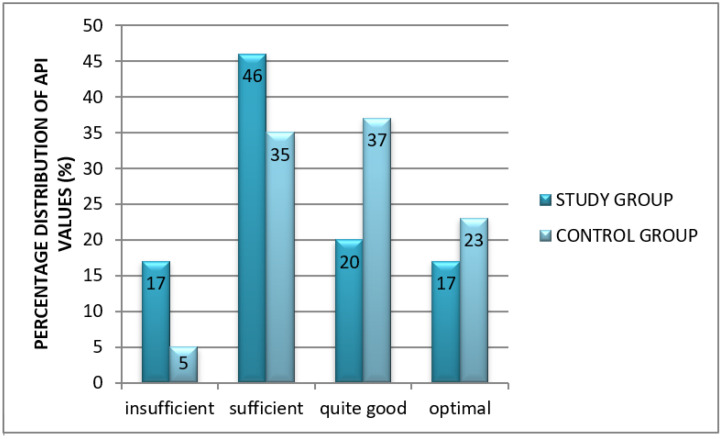
Interpretation of oral hygiene index (API) in both groups.

The analysis of oral hygiene using the approximal plaque index (API) showed that poor hygiene (study group 17% vs. controls 5%) and sufficient hygiene (study group 46% vs. controls 35%) were most often reported in the study group, whereas good hygiene (controls 37% vs. study group 20%) and optimal oral hygiene (controls 23% vs. study group 17%) were more often reported in the control group (Figure 2).

Significantly higher OHI-S was found in the study group (study group 1.55 ± 1.37, range: 3.83–0.17 vs. controls 1.10 ± 1.43, range: 4.49–0). It may be therefore concluded that sufficient oral hygiene was most often reported for CF patients, while good oral hygiene was most often observed in controls.

The mean value of the OHI-S component for debris, i.e., DS-S, was significantly higher in the study group compared to controls (study group 0.90 ± 0.71, range: 2.66–0 vs. controls 0.49 ± 0.59, range: 2.66–0).

**Figure 3:**
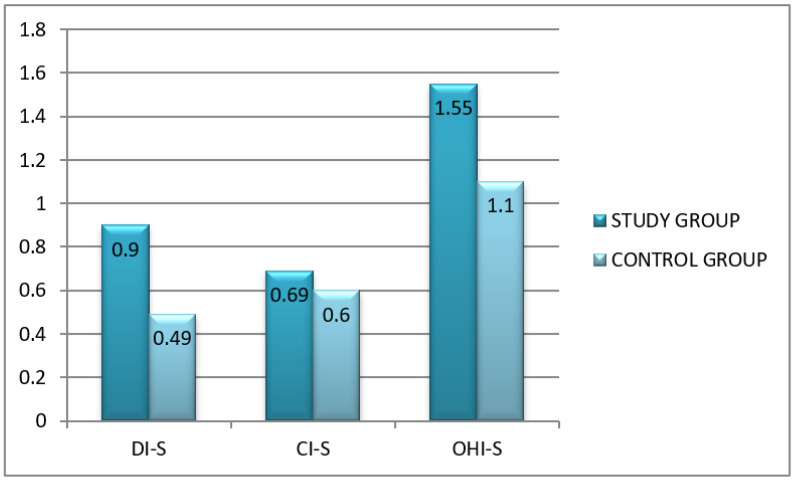
A detailed analysis of oral hygiene using OHI-S index: DI-S—debris; CI-S—calculus; in the study group vs. control group.

No significant differences in the calculus component, i.e., CI-S, were observed (Figure 3).

### 3.3. Questionnaire Results

The results obtained in the survey part of the study indicate that although patients with CF have limited options (due to the intensity of the underlying disease, time-consuming therapy, pharmacotherapy), they do not differ statistically significantly from the responses of patients in the control group with regard to most of the questions regarding daily hygiene habits connecting with using additional products to maintain proper oral hygiene. Only a trend of statistical significance was observed with regard to the additional hygiene equipment used and the frequency of follow-up visits in favor of patients with CF (Table 1).

### 3.4. Community Periodontal Index of Treatment Needs (CPITN)

The CPITN was used to assess periodontal health and treatment needs in both groups.

A healthy periodontium (CPI-0) was most often observed in sextant 13–23 (n = 26; 65%) in the CF group, and sextant 24–27 in the control group (n = 31; 77.5%). Moreover, a lower number of healthy sextants was found in the study group compared to controls, regardless of their location.

Gingival bleeding (CPI-1) was more common in all sextants except for teeth 44–47 (n = 7; 17.5% vs. n = 11; 27.5%) and 43–33 (n = 10; 25% vs. n = 14; 35%) in the study group vs. controls. An increased prevalence of gingival bleeding in sextant 24–27 (n = 19; 47.5% vs. n = 8; 20%) was reported in CF patients.

Calculus (CPI-2) was more often detected in the mandible compared to maxilla in both groups.

Periodontal pockets 4–5 mm (CPI-3) were found only in CF patients, mainly in the anterior teeth of the mandible (43–33) (n = 4; 10%). No pockets > 6 mm (CPI-4) were found in any of the groups. One patient in the CF group was excluded from the study due to edentulism (Table 2 and Table 3).

It was found based on the analysis of treatment needs (TN) that home care (TN-0) and professional instructions on proper oral hygiene (TN-1) were more often needed in the control group compared to CF patients (TN-0: n = 11; 27.5% vs. n = 8; 20.5%. TN-1: n-18; 45% vs. n = 11; 28.2%). In addition to instructions on proper oral hygiene, patients with CF more often required professional preventive treatment in a dental office, such as scaling, cervical and crown polishing, fluoride varnish application (TN-2) (n = 20; 51.3% vs. n = 11; 27.5%).

No need for complex periodontal treatment (TN-3) was reported in any of the groups (Table 4).

## 4. Discussion

There are only a few publications discussing oral hygiene of Cystic Fibrosis patients available [5,9,11,14]. Dąbrowska et al. [5] observed good oral hygiene in that group of patients that was assessed on the basic of OHI-S. However, our observations proved that oral hygiene in that group of patients was satisfactory. Differences in the obtained results may be caused by the number of participants included in the study, patients’ knowledge on the importance of maintenance of proper oral hygiene as well as their knowledge of how to perform oral hygiene procedures properly.

Pawlaczyk-Kamieńska et al.’ [14] results show a poor status of the teeth and extensive spread of dental plaque in adult patients with cystic fibrosis. In patients with cystic fibrosis, there is observed a significant accumulation of dental plaque and no enhanced immune response observed in periodontal tissues. There is no correlation between the amount of periopatological subgingival condition of the gums, which may be explained by a pharmacological decrease in pathogenic potential of plaque (biofilm microorganisms) in these group of patients [14,15]. These results confirm the authors’ observation. It was found based on the analysis of treatment needs that home care and professional instructions on proper oral hygiene were more often needed in the control group compared to CF patients. A healthy periodontium was most often observed in the CF group.

Aps et al. [16] assessed the presence of dental plaque in patients diagnosed with Cystic Fibrosis. They did not observe any statistically significant differences between the examined groups. The use of long-term antibiotics and pancreatic enzymes may converse some protection against the development and progression of dental caries in patients with Cystic Fibrosis.

Aps et al. [17] showed that calculus and gingival bleeding were significantly less common in homozygous CF patients vs. healthy individuals who were carriers of the mutated CFTR gene. Similar results were obtained when comparing CFTR mutation carriers with controls.

A nationwide study conducted by Banach in Poland in 1995 [18] showed that 6.45% of 18-year-old adolescents with CF presented with dental calculus compared to 19.36% in the control group. The data were compared with 18-year-old Polish adolescents, 44.7% of whom had dental calculus. Gingival bleeding was found in 54.55% of adult CF patients, in the absence of calculus and gingival pockets. The following periodontal changes were detected in the control group: gingival bleeding (18.18%), dental calculus (63.64%), and pockets 4–5 mm (18.18%). In their study in CF patients, Olejniczak et al. [19,20] distinguished a group of adult patients, 45.5% of whom had healthy periodontium, while no patients with healthy periodontium were found in the control group. Moreover, no periodontal changes in the form of increased proportion of patients with dental calculus or periodontal pockets 4–5 mm (CPI = 3) and >6 mm (CPI = 4) were found. Dental calculus and pockets 4–5 mm were found in 63.64% and 18.18% of controls, respectively. No periodontal pockets >6 mm (CPI = 6) were reported by Olejniczak [20].

Our study confirmed the lack of periodontal pockets >6 mm (CPI = 4) in CF patients. Periodontal pockets 4–5 mm (CPI-3) were found in patients with CF, mainly in the anterior mandibular teeth (43–33) (n = 4; 10%).

However, the percentage of subjects with a healthy periodontium was higher in the control group compared to CF patients (CPI = 0 27.5% vs. 17.95%). No differences were found in the presence of dental calculus and gingival bleeding between the two groups. The differences between our findings and the above discussed reports of other authors may result from different sample sizes and the choice of controls.

Olejniczak et al. [20] showed that 54.55% of adult CF patients and 100% of controls had treatment needs (TN). In our study, CF patients significantly more frequently required preventive treatments, including professional removal of dental calculus (TN = 2) (n = 20; 51.3% vs. n = 11; 27.5%).

In their study, Marcinkowski et al. [1] emphasized the presence of severe and moderate periodontitis in patients with chronic respiratory diseases after lung transplantation. In this group, patients with CF had moderate periodontitis. However, direct comparison between these studies and our findings is not possible as the cited authors assessed periodontitis based on the definition according to Page and Eke [21], whereas CPITN was used in our study. Despite the different indices used, we found moderate periodontitis in the group of patients with cystic fibrosis.

The habit of breathing through the mouth, typical of chronic respiratory diseases, as well as the inflammation of marginal periodontium, which is related, among other things, to inhaled medications, may contribute to reduced quality of hygienic procedures performed by CF patients. This is associated with pain and gingival bleeding during tooth brushing. Poor cleaning of crowded teeth results in dental plaque accumulation, which increases the already existing gingivitis and contributes to dental caries [20,21].

Preventive care for patients with CF should focus on specialized dietary counseling, oral hygiene instruction, and increased exposure to topical fluorides [22]. Calorie-rich alternatives to fermentable carbohydrates include meats, nuts, and cheeses, along with a balanced diet of fruits and vegetables. Frequency of eating should also be considered and may be attended by a reference to increase the frequency of toothbrushing with fluoride toothpaste. In addition, preventive guidance should be provided, as patients with CF are susceptible to calculus formation due to the higher calcium and phosphorus content of their saliva [23,24].

## 5. Conclusions

In conclusion, the obtained API and OHI-S values in adult CF patients were indicative of satisfactory oral hygiene.

Periodontal treatment needs assessed based on the CPITN index in patients with CF indicated the need for professional prophylaxis procedures, which require the implementation of permanent dental care for this group of patients.

An interdisciplinary dental care to support oral health could be recommendable in individuals with chronic respiratory diseases such as Cystic Fibrosis.

## Figures and Tables

**Table 1:** Results of questionnaire analysis.

Question		Answer	Group				χ^2^	*p*
			Study		Control			
			N	%	N	%		
When have you started to take care of oral cavity hygiene		as a teenager	33	82.5%	31	77.5%	0.313	0.576
		in my 20’s	7	17.5%	9	22.5%		
How long does it take you to brush your teeth?		1 min	5	12.5%	6	15,0%	0.808	0.668
		2 min	20	50.0%	16	40.0%		
		more than 2 min	15	37.5%	18	45.0%		
What kind of toothbrush do you use?	electronic	no	35	87.5%	32	80.0%	0.827	0.363
		yes	5	12.5%	8	20.0%		
	manual	no	5	12.5%	8	20.0%	0.827	0.363
		yes	35	87.5%	32	80.0%		
	hard	no	33	82.5%	32	80.0%	0.082	0.775
		yes	7	17.5%	8	20.0%		
	medium	no	15	37.5%	19	47.5%	0.818	0.366
		yes	25	62.5%	21	52.5%		
	soft	no	32	80.0%	29	72.5%	0.621	0.431
		yes	8	20.0%	11	27.5%		
What type of the toothpaste do you use?	whitening	no	28	70.00%	27	67.5%	0.058	0.809
		yes	12	30.00%	13	32.5%		
	herbal	no	37	92.5%	35	87.5%	0.556	0.456
		yes	3	7.5%	5	12.5%		
	for dentinal hypersensitivity	no	37	92.5%	37	92.5%	<0.001	1.000
		yes	3	7.5%	3	7.5%		
	with fluoride in concentration 1000–1400 ppm	no	20	50.0%	21	52.5%	0.050	0.823
		yes	20	50.0%	19	47.5%		
Do you use any other additional products to maintain proper oral hygiene?	none		10	25.0%	16	40.0%	6.870	0.076
	toothpick		6	15.0%	8	20.0%		
	chewing gum		9	22.5%	11	27.5%		
	dental floss		15	37.5%	5	12.5%		
Do you use mouthwash?	no		27	67.5%	32	80.0%	1.614	0.204
	yes		13	32.5%	8	20.0%		
Do you brush your tongue?	no		17	42.5%	13	32.5%	0.853	0.356
	yes		23	57.5%	27	67.5%		
How often do you visit a dentist?	very rarely		3	7.5%	8	20.0%	6.247	0.044
	only in case of pain		17	42.5%	22	55.0%		
	regularly		20	50.0%	10	25.0%		

**Table 2:** An analysis of periodontal health based on the CPITN index—study group.

Dental Sextants	CPI-0 Healthy	CPI-1 Bleeding	CPI-2 Calculus	CPI-3 Pockets 4–5 mm	CPI-4 Pockets > 6 mm	Excluded
	n (%)	n (%)	n (%)	n (%)	n (%)	n (%)
17–14	17 (42.5%)	18 (45%)	4 (10%)	0 (0%)	0 (0%)	1 (2.5%)
13–23	26 (65%)	11 (27.5%)	2 (5%)	0 (0%)	0 (0%)	1 (2.5%)
24–27	16 (40%)	19 (47.5%)	4 (10%)	0 (0%)	0 (0%)	1 (2.5%)
34–37	18 (45%)	7 (17.5%)	11 (27.5%)	3 (7.5%)	0 (0%)	1 (2.5%)
43–33	9 (22.5%)	10 (25%)	16 (40%)	4 (10%)	0 (0%)	1 (2.5%)
47–44	18 (45%)	7 (17.5%)	12 (30%)	2 (5%)	0 (0%)	1 (2.5%)

**Table 3:** An analysis of periodontal health based on the CPITN index—control group.

Dental Sextants	CPI-0 Healthy	CPI-1 Bleeding	CPI-2 Calculus	CP-3 Pockets 4–5 mm	CP-4 Pockets > 6 mm	Excluded
	n (%)	n (%)	n (%)	n (%)	n (%)	n (%)
17–14	19 (47.5%)	15 (37.5%)	6 (15%)	0 (0%)	0 (0%)	0 (0%)
13–23	30 (75%)	8 (20%)	2 (5%)	0 (0%)	0 (0%)	0 (0%)
24–27	31 (77.5%)	8 (20%)	1 (2.5%)	0 (0%)	0 (0%)	0 (0%)
34–37	30 (75%)	8 (20%)	2 (5%)	0 (0%)	0 (0%)	0 (0%)
43–33	13 (32.5%)	14 (35%)	13 (32.5%)	0 (0%)	0 (0%)	0 (0%)
47–44	28 (70%)	11 (27.5%)	1 (2.5%)	0 (0%)	0 (0%)	0 (0%)

**Table 4:** An analysis of treatment needs (TN) based on the CPITN index in the study and control group.

Periodontal Treatment Need Category	Study Group n = 40		Control Group n = 40	
	n	%	n	%
TN-0	8	20.5	11	27.5
TN-1	11	28.2	18	45
TN-2	20	51.3	11	27.5
TN-3	0	0	0	0
	Chi^2^ *p* = 0.092			

## Data Availability

The data presented in this study are available on request from the corresponding author.
